# Advances in the application of CRISPR technology in pathogen detection: amplification-based and amplification-free strategies

**DOI:** 10.3389/fcimb.2025.1645699

**Published:** 2025-11-03

**Authors:** Xiaoxing Zhou, Chao Ye, Mengru Xie, Yan Wei, Yilian Zhao, Xinchu Liu, Jinghui Ma, Jilin Qing, Zhizhong Chen

**Affiliations:** ^1^ School of Clinical Medicine, Guilin Medical University, Guilin, China; ^2^ Precision Joint Inspection Centre, The People’s Hospital of Guangxi Zhuangzu Autonomous Region and Guangxi Academy of Medical Sciences, Nanning, China; ^3^ The First Clinical Medical College of Guangxi Medical University, Nanning, China; ^4^ Graduate College, Guangxi University of Chinese Medicine, Nanning, China; ^5^ Center for Reproductive Medicine and Genetics, The People’s Hospital of Guangxi Zhuangzu Autonomous Region and Guangxi Academic of Medical Sciences, Nanning, China

**Keywords:** CRISPR technology, pathogen detection, amplification-based CRISPR, amplification-free CRISPR, isothermal amplification, sensor technology

## Abstract

CRISPR technology, with its high specificity and programmability, has become an important tool for the detection of human pathogens. The timely and accurate detection of pathogens is crucial for public health. In recent years, significant progress has been made in the application of CRISPR technology for pathogen detection. However, several challenges remain, including detection sensitivity, specificity, and operational convenience. This review summarizes the latest advances in CRISPR technology for pathogen detection, with a focus on the principles and performance comparisons of amplification-based CRISPR (such as those combined with isothermal amplification techniques like RPA and LAMP) and amplification-free CRISPR (such as cascade CRISPR, sensor technologies, and digital droplet CRISPR). It also discusses their applications in pathogen detection. In addition, the article analyzes the advantages and limitations of CRISPR detection technology and looks forward to future development trends, providing a theoretical basis for the optimization of rapid diagnostic techniques for pathogens.

## 1 Introduction

**Figure 1:**
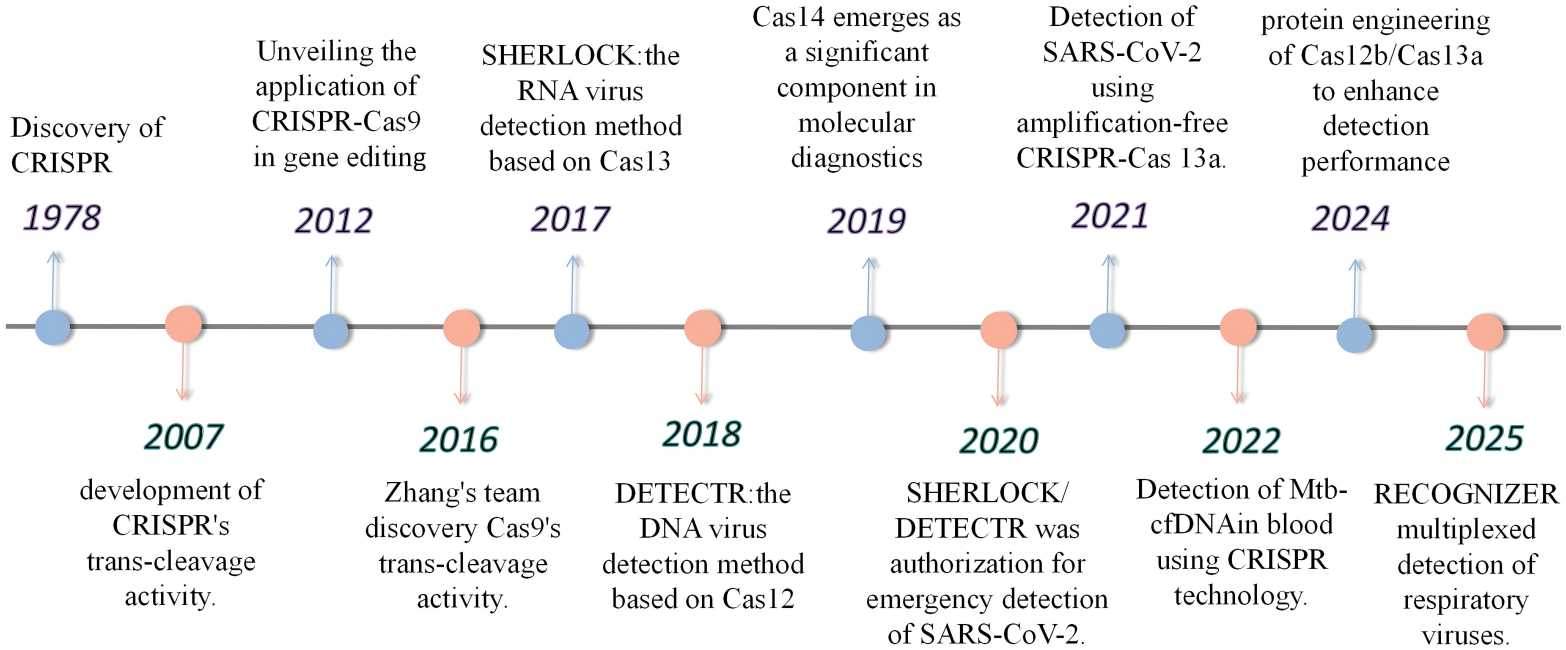
Schematic overview of the development and core components of CRISPR detection technology.

In the field of pathogen detection, various techniques have their own characteristics. Microbial culture, as the “gold standard” for laboratory detection, is capable of determining microbial viability and identifying low-abundance microbes. However, it is time-consuming (usually taking 2–10 days) (Shetty et al., 2003), and demands high technical skills and biosafety precautions, making it unsuitable for rapid detection needs. In immunological detection, antigen tests are mainly used in the early stages of infection, while antibody tests have a relatively low positive rate in the initial infection phase (approximately 27%-41%) (Deeks et al., 2020), which can increase to 78%–88% in the second to third week of infection. Traditional PCR-based detection techniques (such as qPCR) offer high sensitivity but require a long detection time and have high demands for equipment and technical personnel, making them unsuitable for point-of-care testing (POCT) during pathogen outbreaks. In recent years, isothermal amplification technologies (such as RPA and LAMP) have been successfully established to work at a constant temperature to eliminate the dependence on thermal cycles. However, isothermal amplification technologies usually have some disadvantages, such as non-specific. In recent years, the CRISPR (Clustered Regularly Interspaced Short Palindromic Repeats) system and its associated Cas proteins have been widely applied in the field of molecular diagnostics. Since its discovery in 1987, the CRISPR system has been identified as an adaptive immune mechanism in bacteria (Doudna and Charpentier, 2014). It enables recognition of specific nucleic acid sequences and achieving pathogens detection. CRISPR comprises six types (I–VI); II, V and VI are well characterized for gene editing and pathogen detection (Makarova et al., 2015; Shmakov et al., 2017) (**Figure 1**).

CRISPR detection methods are primarily categorized as amplification-based and amplification-free. Amplification-based approaches offer high sensitivity and specificity while requiring less complex instrumentation, making them an important advancement in molecular diagnostics. The application of CRISPR technology in the detection of various pathogens has also become increasingly widespread, including viruses (Miao et al., 2023), bacteria (Wu et al., 2021b), mycoplasma (Li et al., 2022b; Chen et al., 2022);, etc. Amplification-free CRISPR has gradually gained attention. Compared with traditional amplification-based methods, amplification-free CRISPR can eliminate the amplification step, reducing operational complexity and the risk of potential contamination. Researchers have explored a variety of amplification-free CRISPR detection strategies, which include innovative methods such as cascade CRISPR, sensor devices, and digital droplet CRISPR. These strategies are capable of sensitive pathogen detection without amplification. For example, an amplification-free CRISPR-Cas13a platform for the detection of SARS-CoV-2 was established, which can detect the virus down to 470 aM within 30 minutes, showing the potential of amplification-free strategies in pathogen detection (Shan et al., 2023).

Through continued refinement of CRISPR technology is anticipated to result in more efficient and convenient pathogen detection tools. This review delves into the principles of amplification-based and amplification-free CRISPR technologies, analyzes their advantages and limitations, introduces their applications in pathogen detection, and discusses their future prospects, aiming to offer a comprehensive reference for research in this field.

## 2 The principle of CRISPR immune activation cleavage

The CRISPR system is characterized by its excellent specificity and flexibility, which enables precise gene editing and regulation in a variety of organisms (Fang et al., 2023). It has not only played a significant role in basic research but also demonstrated broad prospects in applied research. In terms of applications, CRISPR technology has been involved in areas such as agriculture (Chen et al., 2019), biological research (Manghwar et al., 2019; Sanjana et al., 2014), environmental monitoring (Zhang et al., 2021), and disease treatment (Shokoohi et al., 2025a). In the field of molecular diagnostics, CRISPR technology has become an important tool.

**Figure 2:**
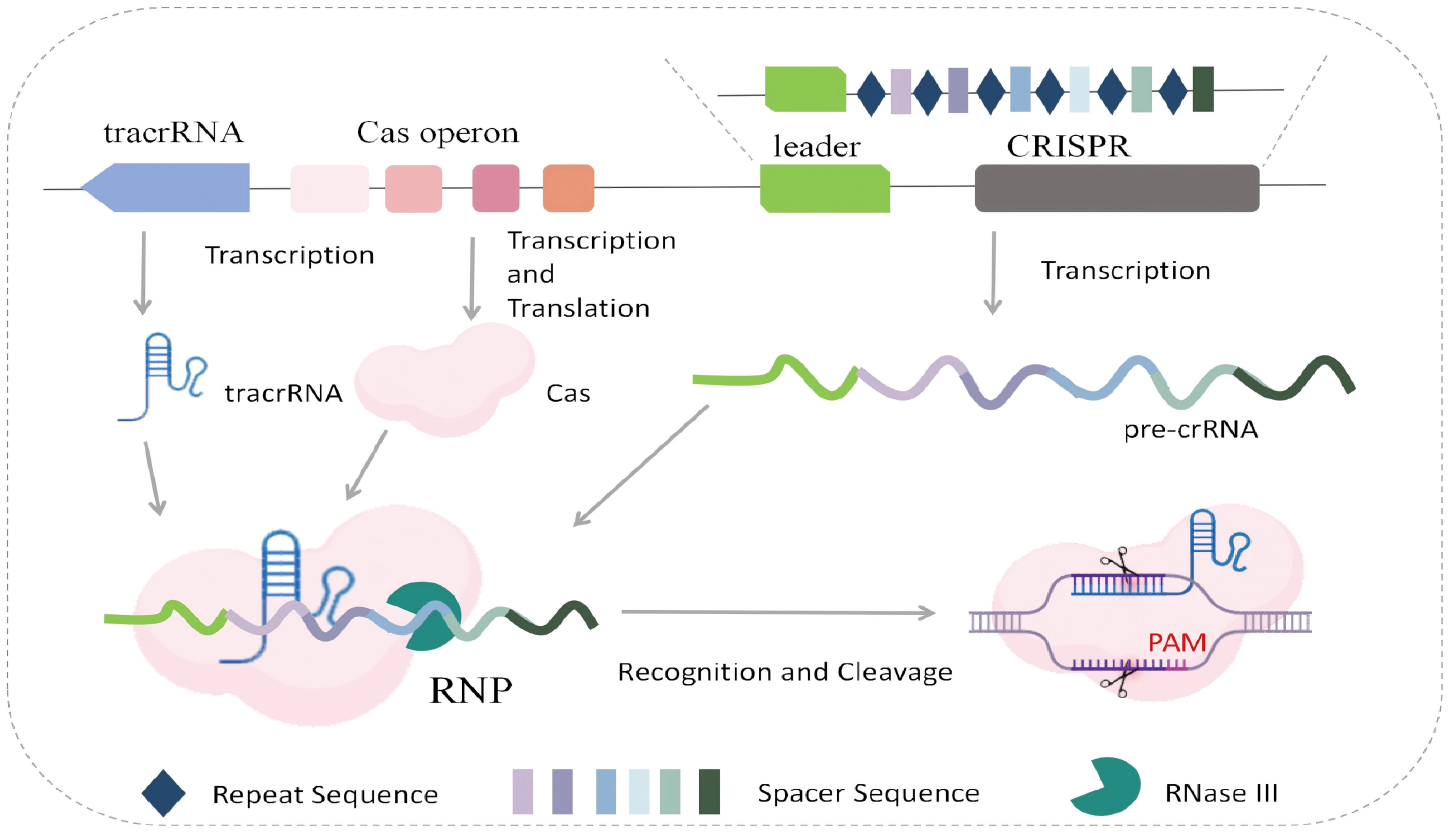
Mechanism of immune activation and nucleic acid cleavage by the CRISPR system.

Taking Cas9 as an example, the immune activation mechanism of the CRISPR system is as follows: The CRISPR locus consists of spacer sequences and repeat sequences. When bacteria are first attacked by foreign material, the CRISPR system inserts a fragment of the foreign material’s gene into the spacer region. Upon re-infection by the same material, the repeat sequences and spacers in the CRISPR locus are transcribed into pre-crRNA, while the tracrRNA gene is transcribed into tracrRNA. Cas proteins (such as Cas9), tracrRNA, and pre-crRNA form an RNP complex. During this process, pre-crRNA matures and combines with tracrRNA to form crRNA. The crRNA guides the Cas protein complex to recognize the spacer sequence and PAM sequence in the target DNA. The Cas protein cleaves the double-strand of the target DNA at a specific position upstream of the PAM sequence, generating double-strand breaks (DSBs). In addition, after cleaving their targets, Cas9 (Hatada et al., 2023), Cas12a (Swarts and Jinek, 2019), and Cas14 (Cas12f) (Aquino-Jarquin, 2019) exhibit non-specific cleavage of single-strand DNA (ssDNA), while Cas13 shows non-specific cleavage activity against single-strand RNA (ssRNA). This phenomenon is known as trans-cleavage activity (**Figure 2**).

### 2.1 Principal of CRISPR detection

Researchers have leveraged the CRISPR-mediated principles of immune activation and cleavage to develop CRISPR-based *in vitro* diagnostic technologies, typically used for pathogen detection. Researchers design specific crRNAs to guide the CRISPR complex to bind and activate the target, thereby specifically cleaving the target and non-specifically cleaving the reporter molecule (a single-stranded nucleic acid fragment). By labeling the reporter molecule with fluorescent signals (such as FAM, ROX, etc.) and quenching groups (such as BHQ), to detection of pathogens. Additionally, biotin and FAM or digoxigenin (dig) can be labeled on the reporter molecule, and detection can be performed using lateral flow assay (LFA) strips. LFA does not require complex instruments, making the detection process more convenient and further expanding the application potential of CRISPR technology in POCT.

Commonly used Cas proteins include Cas9, Cas12a, Cas13, and Cas14 (Zhou et al., 2024a). In addition, other Cas proteins such as CasΦ (Cas12j) (Kang et al., 2024) and Cas14 (Cas12f) (He et al., 2024) have also been applied. Among them, Cas9, Cas12a, and Cas14 (Leung et al., 2022; Qiu et al., 2022) recognize and bind to DNA or RNA, while Cas13 (Abudayyeh et al., 2017; Cox et al., 2017) recognizes and binds to single-stranded RNA. The characteristics of main Cas protein in pathogen detection are shown in **Table 1**.

**Table 1:** Comparison of characteristics of main Cas proteins in pathogen detection.

Characteristic	Cas9	Cas12a	Cas13	Cas14a
target	DNA/RNA	DNA/RNA	RNA	dsDNA/RNA
PAM	NGG	TTTV, etc.	None	None
Trans-cleavage Activity	Non-specific ssDNA	Non-specific ssDNA	Non-specific RNA	Non-specific ssDNA
Sensitivity	medium	high	high	high
Specificity	high	Medium	Medium	Very high
Applicable Scenario	Laboratory research	DNA pathogens	RNA pathogens	SNP/Short ssDNA
Commercialization	Limited	Extensive	Extensive	Limited
Reference	(Chen et al., 2025b; Gao et al., 2021)	(Rananaware et al., 2023; Wang et al., 2023b)	(Granados-Riveron and Aquino-Jarquin, 2021)	(Yang et al., 2025)

The evaluation levels of sensitivity and specificity are derived from a horizontal comparison of the performance parameters of each Cas protein, including key indicators such as the detection limit (LOD) and the ability to recognize single-base mismatches.

### 2.2 Classification of CRISPR detection

The application of CRISPR technology is becoming increasingly widespread in pathogen detection. Based on their application methods, CRISPR technologies can be divided into two major categories: amplification-based CRISPR and amplification-free CRISPR.

Amplification-based CRISPR technology relies on nucleic acid amplification techniques, such as RPA, LAMP, and multiple cross displacement amplification (MCDA), these techniques enhance the detection sensitivity for pathogens detection. For instance, the combination of RPA and CRISPR-Cas12a can achieve rapid detection of Mpox DNA within 30 minutes, and sensitive as low as 1 copy of (Zhao et al., 2023). Similarly, by combining LAMP with CRISPR-Cas12a has also shown good performance in detecting bacterial pathogens (Lee and Oh, 2022).

In contrast, amplification-free CRISPR emphasizes the direct detection of pathogens, which reduces the cumbersome amplification steps and decreases detection time. For instance, a CRISPR-Cas13a-based system can rapidly generate signals by directly binding to RNA targets, and achieving pathogen detection. Studies have shown that this method exhibits good specificity and sensitivity in detecting the HIV-1 virus (Zhang et al., 2025). Moreover, amplification-free CRISPR systems combined with novel sensor technologies (such as gFET, ECL, SERS) can directly monitor targets through signal transduction.

### 2.3 Principal of amplification-based CRISPR detection

Amplification-based CRISPR technology combines isothermal amplification techniques with the CRISPR system. The introduction of isothermal amplification significantly enhances detection sensitivity. Currently, amplification-based CRISPR methods are primarily categorized into two-step and one-step detection assays.

#### 2.3.1 Principle of two-step amplification-based CRISPR detection

In the workflow of two-step amplification-based CRISPR, pathogens are first rapidly amplified using isothermal amplification techniques. After amplification is completed, the amplified products are manually introduced into the CRISPR reaction system.

**Figure 3:**
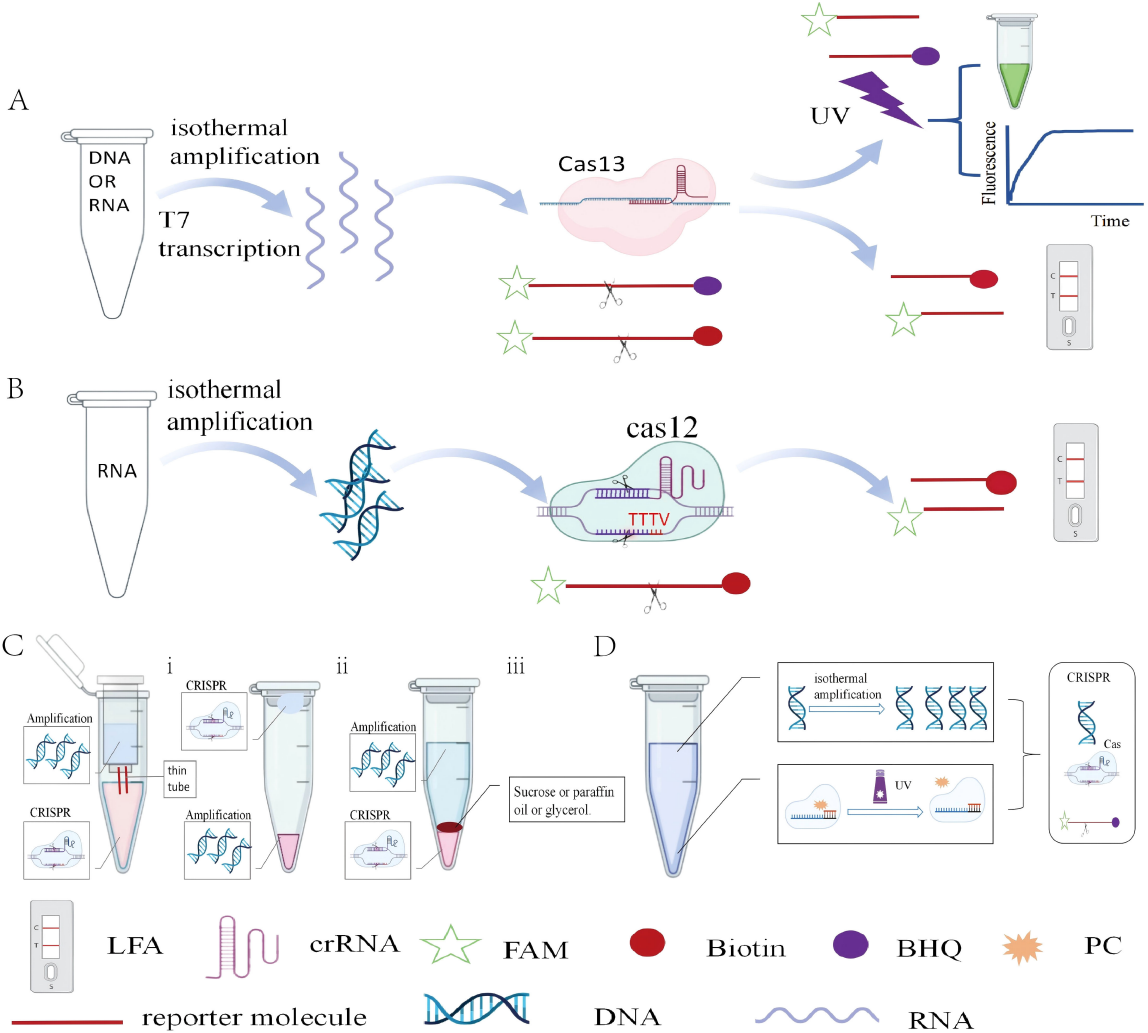
The principle of amplification-based CRISPR **(A)** SHERLOCK: RNA or DNA templates undergo isothermal amplification and T7-mediated reverse transcription, activating the CRISPR-Cas13 system to cleave reporter molecules for fluorescence or lateral flow assay (LFA) detection. **(B)** DETECTR: DNA templates are amplified isothermally to activate the CRISPR-Cas12 system, leading to reporter cleavage and fluorescent signal generation. **(C)** Physically segregated one-pot strategies: (I) Dual-chamber tubes connected by a narrow channel. (ii) Separate amplification (in cap) and CRISPR (in bottom) compartments, mixed by centrifugation. (iii) Layer-based isolation using high-density barriers (e.g., sucrose or mineral oil). **(D)** The photocleavable (PC) linker acts as a silencing factor. Its conjugation to the crRNA keeps the CRISPR system inactive. UV irradiation cleaves the linker, reverses the silencing effect, and activates the CRISPR system.

The main application examples of amplification-based CRISPR technology include SHERLOCK (Kellner et al., 2019) and DETECTR (Broughton et al., 2020). SHERLOCK focuses on the CRISPR-Cas13 system and achieves sensitivity detection by RPA and transcribing DNA into RNA. The core of this process is the enzymatic activity of CRISPR-Cas13, which recognizes target sequences through crRNA and cleaves them after recognition. A significant advantage of this technology is 100% sensitivity and specificity demonstrated in clinical samples (**Figure 3A**). DETECTR is based on the CRISPR-Cas12 system and is primarily used for DNA detection. It combines RPA with CRISPR-Cas12 to achieve detection of DNA (**Figure 3B**). In two-step amplification-based CRISPR detection, the need to manually transfer amplified products into the CRISPR system not only increases operational complexity but also raises the risk of aerosol contamination.

#### 2.3.2 Principle of one-pot amplification-based CRISPR detection

To address these issues, researchers have explored various strategies to integrate amplification and CRISPR reactions into a single process. Currently, the main solutions include physically separating and optimizing reaction conditions to enabling one-pot reactions.

Firstly, the physically separation can be achieved by modifying the reaction tube design. For example, a double-layer reaction tube was designed, where the inner and outer layers are connected by a thin tube (Hu et al., 2022a). The inner layer is used for RPA and after completion, the amplified products are transferred to the outer layer containing the CRISPR reaction system through centrifugation, thus enabling one-pot detection. Additionally, the CRISPR reaction system was placed on the cap of the reaction tube (Sun et al., 2021). After amplification, the two systems are mixed by centrifugation. This optimized method can detect SARS-CoV-2 within 50 minutes, with a detection limit of 1 copy/μL. Secondly, physical separation can also be achieved by adding dense reagents (such as sucrose, mineral oil, and glycerol). A dynamic aqueous multiphase reaction system (DAMR) that exploits sucrose-density differences to compartmentalize two otherwise incompatible reactions in the same tube was developed (Yin et al., 2020). Paraffin was used to separate the RAA and CRISPR-Cas12a systems, ensuring that sufficient RAA amplicons diffuse to activate the CRISPR-Cas12a reaction (Tan et al., 2024). This method achieves a sensitivity of 2.5 × 10 copies/μL (**Figure 3C**).

Researchers have explored various innovative methods to efficiently integrate the two processes. LAMP and Cas12b-both active at 58°C-were combined to enable one-pot CRISPR detection (Gong et al., 2024). It was found that AacCas12b exhibits minimal cis-cleavage but robust trans-cleavage at 30-39°C, based on this, RPA and CRISPR/AacCas12b were integrated in a single tube to create the Rcod system (Lin et al., 2024). Additionally, a one-pot detection method utilizing suboptimal protospacer adjacent motifs (PAMs) for Cas12a, such as VTTV, TCTV, and TTVV, was developed (Lu et al., 2022). Compared to the traditional TTTV PAM, these suboptimal PAMs exhibit lower binding affinity, thereby reducing the cis-cleavage activity of Cas12a. This allows to more effectively accumulate amplified products, providing sufficient substrates for the Cas12a reaction and offering more flexible crRNA design. A photocontrolled one-pot CRISPR workflow was likewise established (Hu et al., 2022b). However, this inevitably entails low sensitivity and mutual interference between amplification and CRISPR. (**Figure 3D**).

### 2.4 Principal of amplification-free CRISPR detection

**Figure 4:**
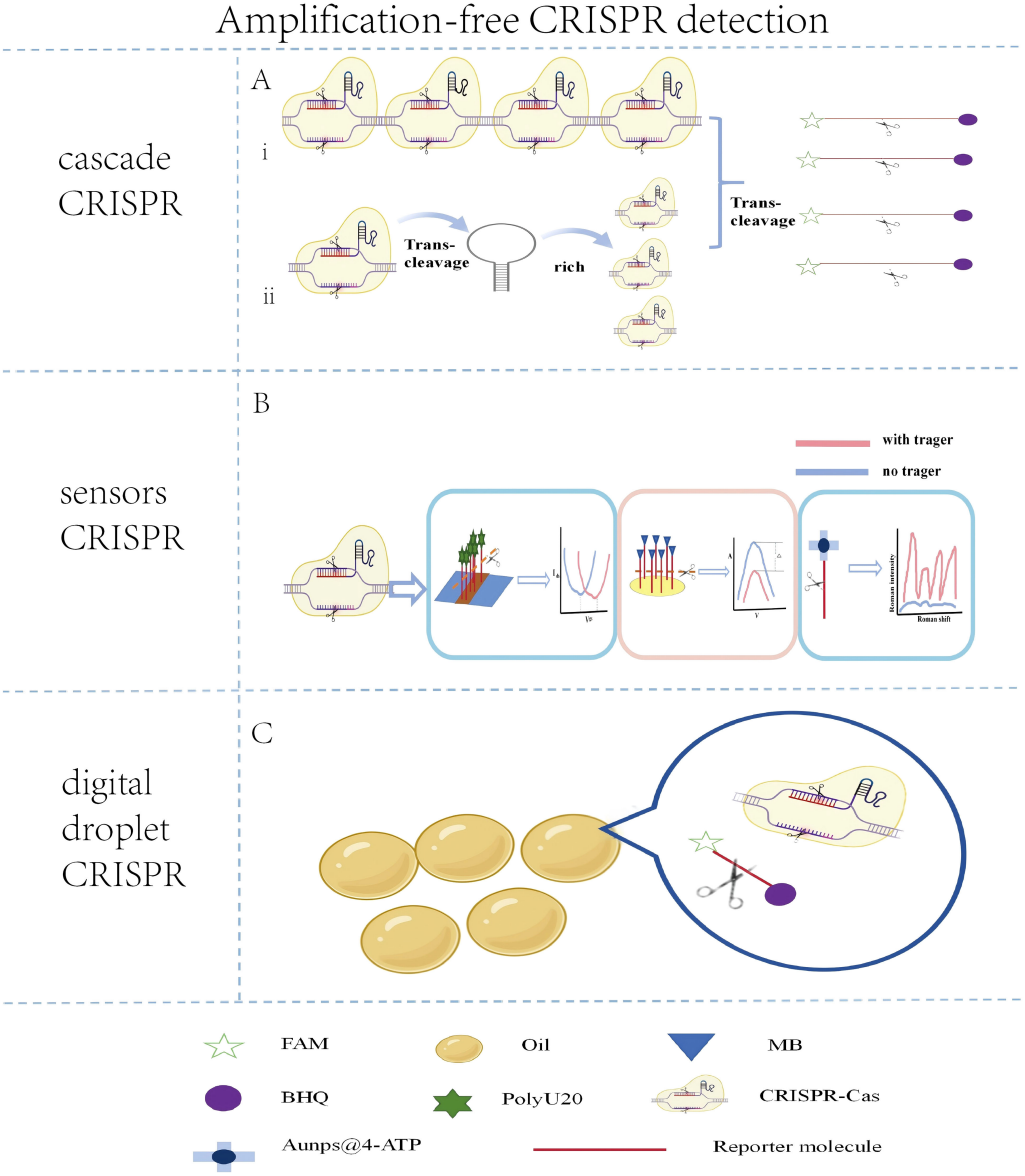
Schematic illustration of key amplification-free CRISPR detection strategies. **(A)** Cascade signal amplification approaches. (I) Multi-crRNA strategy: Simultaneous use of multiple crRNAs targeting a single pathogen enhances Cas protein activation and signal output. (ii) Nucleic acid circuit-based amplification: Autocatalytic reactions, such as the cleavage of a designed U-rich hairpin (URH) structure, generate secondary targets to activate additional Cas proteins for signal enhancement. **(B)** Sensor-integrated CRISPR platforms. Schematics of biosensors coupled with CRISPR for direct signal transduction, including gFET, ECL, and SERS sensors. **(C)** Digital droplet amplification-free CRISPR utilizes oil-phase droplets to achieve single-molecule detection.

Amplification-free CRISPR technology is an emerging detection method whose key advantage lies in its ability to detection of pathogens without relying on traditional nucleic acid amplification techniques (**Figure 4**).

#### 2.4.1 Cascade CRISPR detection

In the CRISPR reaction system, crRNA scans and recognizes specific sequences to activate the Cas protein. The more crRNA species present, the more Cas proteins are activated, thereby enhancing the trans-cleavage activity. In amplification-free CRISPR detection strategies, to activate more Cas proteins and improve detection sensitivity, researchers have employed cascade methods, primarily including multi-protease cascade signal amplification and nucleic acid circuit signal amplification.

The multi-protease cascade signal amplification method mainly involves designing multiple crRNAs on the target. The detection limit for single crRNA was found to be around 80.52 pM, whereas for triple or quadruple crRNAs, the detection limit was between 6.92 and 1.25 pM. The detection limit for multiple crRNAs was 6 to 64 times higher than that for single crRNA (Zeng et al., 2022). Four crRNAs were designed on the target sequence, achieving a detection sensitivity of 0.16 pM (Yang et al., 2024). Similarly, a CRISPR-Cas12a system with multiple crRNAs was utilized to detect microsporidians (Zhang et al., 2024), which not only saved time but also reduced the risk of aerosol contamination, offering a new approach for detecting other pathogens.

Nucleic acid circuit signal amplification involves specially designed nucleic acid fragments to activate a secondary CRISPR reaction. A U-rich hairpin (URH) structure was be designed to replace the reporter molecule, with the loop part rich in uracil preferred for Cas13a cleavage and the stem part containing complementary bases to ensure the stability of the URH structure. In the presence of the target, the CRISPR system is activated, the URH structure is cleaved, and the released stem part serves as a target for secondary cleavage. Without amplification, this method can detect 100 copies/μL of SARS-CoV-2 within 5 minutes (Zeng et al., 2024). However, the method requires operation at low temperatures and is prone to false-positive results. Additionally, the DSAC detection system was developed by introducing circular nucleic acid sequences into the CRISPR reaction system (Zhou et al., 2024c). These sequences are cleaved into double strands by the trans-cleavage activity of Cas12a, thereby activating the CRISPR system. Furthermore, an amplification-free collateral cleavage-enhanced CRISPR-CasΦ method (TCC) was established, enabling pathogen detection with a sensitivity as low as 1.2 CFU/mL within 40 minutes (Chen et al., 2025a). This approach can also be extended for miRNA detection (**Figure 3A**).

However, Cascade CRISPR also bring many challenges. First, the complexity of crRNA design is increased. Second, nucleic acid circuit signal amplification is prone to false-positive results, which can affect the accuracy of detection.

#### 2.4.2 Sensors combined with CRISPR detection

At present, amplification-free CRISPR technology can also use more sensitive sensor devices to efficiently output weak signals. Among them, graphene field-effect transistors (gFET), electrochemical biosensors (ECL), and surface-enhanced Raman spectroscopy (SERS) are the three main types of sensor devices.

gFET achieve ultrasensitive nucleic acid detection by manipulating the conductivity of graphene. gFET consists of a graphene channel, source and drain electrodes connected to the graphene channel, and a gate electrode (Sun et al., 2024). The Cas13a was utilized in combination with a gFET, successfully enabling the detection of SARS-CoV-2 at the aM level within 30 minutes at 37°C (Li et al., 2022c). In this system, negatively charged RNA reporter molecules (such as PolyU20) are immobilized on the surface of the gFET via a molecular linker 1-pyrenebutyrate succinimide ester (PBASE). Upon activation of CRISPR Cas13a, the reporter molecules are cleaved, leading to a reduction in the electron-doping effect on the graphene surface and a positive shift in the charge neutrality point voltage (VCNP). The quantitative detection of target nucleic acids is achieved by measuring the change in VCNP. Further optimized this technology by designing reporter molecules (RP) with stronger electron-doping effects, significantly enhancing the specificity and sensitivity of detection (Sun et al., 2023). However, the stability of graphene in liquid environments remains a challenge. Although hydrophobic treatments (such as OTS coating) can enhance stability, the long-term stability of graphene in complex clinical samples still needs to be verified.

ECL are commonly used signal output devices in amplification-free CRISPR detection, consisting of an electrode system, an electrolyte solution, and an electrochemical indicator. The electrochemical CRISPR (E-CRISPR) method that uses methylene blue (MB)-modified reporter molecules. In the presence of the target, the trans-cleavage of the reporter molecules releases MB, altering the redox reactions on the electrode surface and generating a current signal. This method successfully detected HPV-16, PB-19, and TGF-β1 with a detection limit at the nanomolar (nM) level (Dai et al., 2019). Additionally, methylene blue (MB) and ferrocene (Fc) were employed as electrochemical indicators to detect the S gene and ORF1ab gene of SARS-CoV-2, utilizing the MB current signal in the negative potential range and the Fc current signal in the positive potential range (Kashefi-Kheyrabadi et al., 2023). Electrochemical biosensors offer sensitivity, rapid response, and ease of operation, making them suitable for rapid diagnosis and large-scale screening.

SERS is a spectroscopic technique based on the enhancement of Raman signals by metal nanostructures. SERS technology was utilized for the detection of SARS-CoV-2 without amplification, achieving a detection limit of 1 fM (Liang et al., 2021). This method involves attaching reporter molecules modified with biotin and thiol groups to streptavidin-coated magnetic beads and silver nanoparticles@4-ATP to form SERS probes. After activation of the CRISPR-Cas12 system, the reporter molecules are cleaved, causing the silver nanoparticles@4-ATP to dissociate and resulting in a decrease in Raman signals, with the signal change being proportional to the viral concentration. In addition to 4-ATP, commonly used Raman reporter molecules include 4-mercaptobenzoic acid (4-MBA). As demonstrated in one study, 4-MBA was conjugated to gold nanoparticles for this purpose (Ma et al., 2023). Furthermore, the operation process was significantly simplified by employing centrifugation or polyether sulfone (PES) membrane filtration for signal detection (Liang et al., 2021).

While gFET, ECL, and SERS technologies offer high sensitivity and specificity for amplification-free CRISPR detection, they differ in cost, stability, and operational complexity. gFET sensors require improvement in stability and remain in the laboratory research and development stage, making accurate estimation of their practical large-scale production costs challenging. ECL sensors strike a favorable balance between cost and performance, with relatively low instrument prices (approximately $5,000–$20,000), yet they still face background interference issues that require further optimization to improve the signal-to-noise ratio. In contrast, despite its outstanding detection performance, the high equipment acquisition cost of SERS technology (ranging from about $50,000 to $200,000 depending on configuration) limits its large-scale application in clinical settings. Future advancements in materials science and nanotechnology will drive the development of more stable, cost-effective, and user-friendly sensor devices, thereby laying a critical foundation for the practical application and widespread adoption of amplification-free CRISPR detection technology. (**Figure 3B**).

#### 2.4.3 Digital droplet CRISPR detection

Digital droplet CRISPR technology is an emerging method for pathogen detection, utilizing microdroplet techniques to achieve quantitative detection of pathogens at the single-molecule level. The core of digital droplet CRISPR technology lies in the integration of the CRISPR system with droplet digital technology. The basic principle of droplet technology is to use microfluidics to divide sample liquids into micrometer-sized droplets, each of which can independently undergo a reaction. STAMP digital CRISPR-Cas13a technology to achieve quantitative detection of HIV-1 RNA, with a detection limit of 2000 copies/ml (Nouri et al., 2023). This technology uses commercial polycarbonate track-etched (PCTE) membranes to digitally partition the reaction mixture, utilizing its high-density and uniform-sized pores to divide the sample into tiny reaction units. Further proposed by combining the CRISPR system and target nucleic acids with an oil phase (90% isopropyl palmitate and 10% Abil EM 180) to generate polydisperse droplets with diameters of 10-100 μm through simple vertexing, with 90% having diameters less than 50 μm (Xue et al., 2023). This method significantly reduces the difficulty of droplet generation, eliminating the need for complex microfluidic devices and enhancing the convenience and accessibility of detection, making it particularly suitable for POCT and pathogen detection in resource-limited environments. By combining sample micro-segmentation with the CRISPR system, it may become a valuable tool for crisis response as the technology matures.(**Figure 3C**).

In brief, CRISPR-based nucleic acid detection has develop from amplification-dependent to amplification-free approaches. The two strategies differ in sensitivity, specificity, limitations, and strengths, as summarized in **Table 2**.

**Table 2:** Comparison of amplification-based and amplification-free CRISPR technologies.

CRISPR		Sensitivity	Specificity	Time	strengths	limitations	reference
amplification- based CRISPR	RPA+ CRISPR	1.9 copies/μL,	100%	60-150min	Mature technology, simple primer design	Requires amplification, complex operation, contamination risk	(Zhou et al., 2024b, Wu et al., 2024)
	LAMP+ CRISPR	1 copy/μL	100%	<60 min	Widely applied	Complex primer design	(Lei et al., 2022, Zhang et al., 2023b)
Amplification- Free CRISPR	Cascade CRISPR	84 CFU/mL	100%	30 min	Improved specificity	Complex crRNA design, prone to false positives	(Bao et al., 2023)
	Sensors CRISPR	2.42 × 10^−18^ nM	98.60%	5 min	Efficient signal output	Signal instability, background interference, expensive equipment	(Wang et al., 2025)
	Digital Droplet CRISPR	100 aM,10 aM	100%	30 min	quantification	High equipment cost, limited accessibility	(Shinoda et al., 2021)

## 3 Applications of CRISPR in human pathogen detection

**Figure 5:**
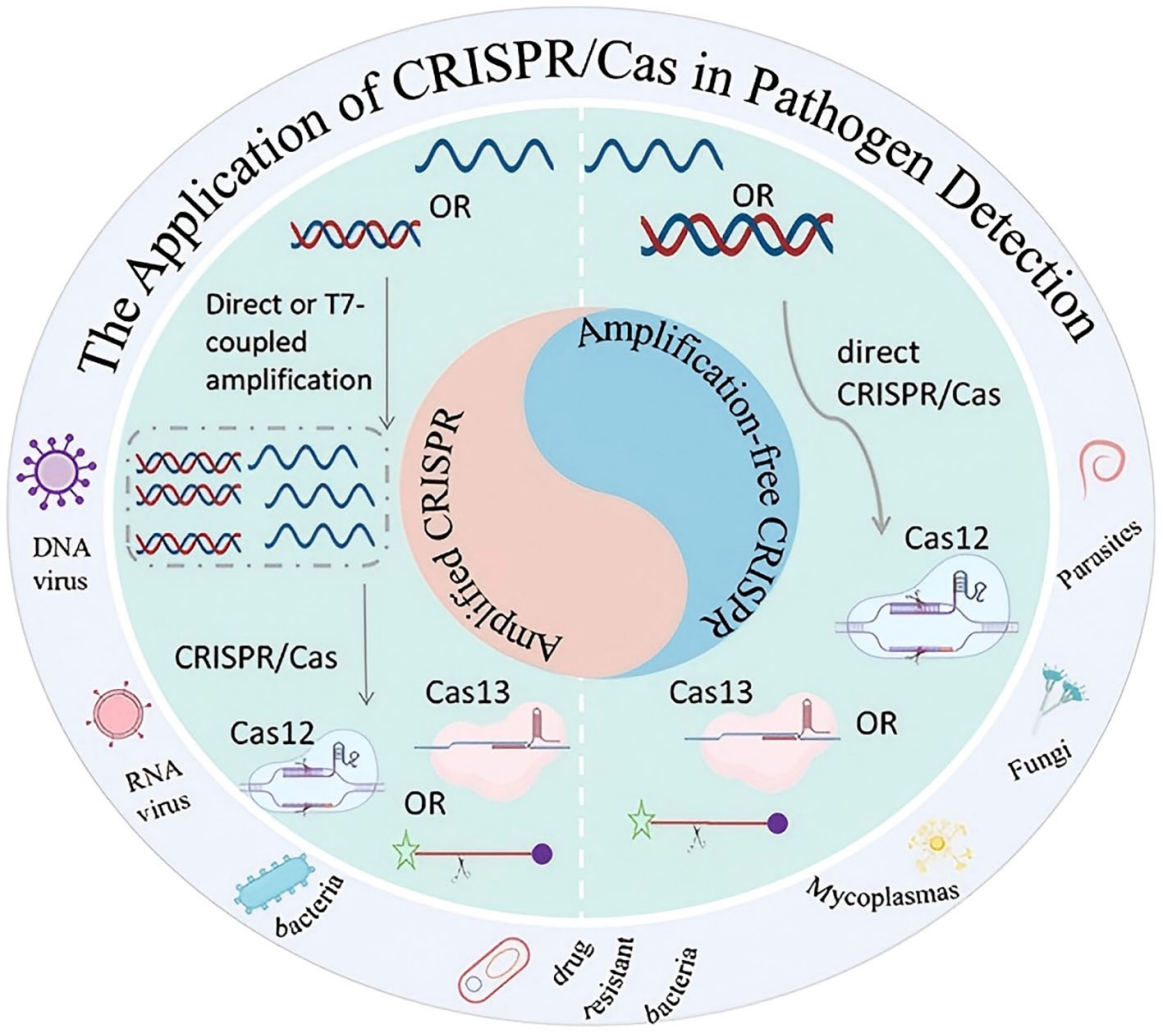
Application of CRISPR technology in pathogen detection: amplification and amplification-free strategies.

CRISPR technology serves as a molecular tool for detecting various pathogens. as shown in **Figure 5**.

### 3.1 Viral pathogens

The outbreak of the SARS-CoV-2 pandemic, along with the persistent threat of other viral diseases, has made the development of rapid, sensitive, and specific viral detection methods particularly crucial. CRISPR systems have been successfully applied for rapid detection of various viruses, including influenza virus (Yoshimi et al., 2022), respiratory syncytial virus (Khamwut et al., 2025), SARS-CoV-2 (Nouri et al., 2021), and its variants (Y. Liang et al., 2021), which holds significant practical implications for responding to public health emergencies.

#### 3.1.1 RNA viruses

Viruses are simple-structured, minuscule pathogens (Taylor and Leiman, 2020). During infection, they spread rapidly and widely, and are prone to mutation, especially RNA viruses, a trait that often affects the efficacy of vaccines and drug treatments. In 2020, the SHERLOCK detection technology was optimized, leading to the establishment of a one-pot CRISPR-Cas12b SARS-CoV-2 detection method known as STOPCovid.v1 (Joung et al., 2020). This method integrates magnetic bead extraction, LAMP and LFA, enabling virus detection within one hour. The sensitivity and specificity were 93.1% and 98.5%, respectively, as tested on 202 positive and 200 negative samples. In 2021, an amplification-free CRISPR-Cas13a SARS-CoV-2 detection method was developed, which enabled direct quantification of viral concentration using a mobile phone microscope (Fozouni et al., 2021). This technique, which employs triple crRNA to directly recognize and cleave reporter molecules, can detect the virus within 30–40 minutes, with a sensitivity of 30 copies/μL. The method established is a rapid POCT technology that could be adapted for portable detection, reducing reliance on specialized laboratories and personnel. SATORI (Iida et al., 2023) is an automated digital droplet CRISPR detection platform that can detect SARS-CoV-2 and influenza A/B viruses in just 9 minutes, with a sensitivity of 6.5 aM after magnetic bead enrichment. Mpox, a zoonotic virus (also known as monkeypox virus), has prompted researchers to develop a CRISPR-Cas12a-based detection system that can simultaneously detect mpox virus and other Ortho poxviruses, highlighting its importance in rapidly responding to outbreaks (Singh et al., 2023). Hepatitis D virus (HDV) can accelerate the progression of chronic hepatitis B virus infection and poses a health burden to patients. A CRISPR detection method based on RT-RAA was established, demonstrating a sensitivity of 10 copies/μL in positive samples. Ebola virus infection causes hemorrhagic fever, a disease with an extremely high mortality rate, for which there is currently no specific treatment. An amplification-free Cas-Roller method, which can detect Ebola virus down to 291 aM within 40 minutes at 37°C (Hang et al., 2022). In summary, the application of CRISPR technology, especially the Cas13a system, is gradually changing the way RNA viruses are detected, providing new solutions for viral detection.

#### 3.1.2 DNA viruses

In the detection of DNA viruses, the CRISPR-Cas12a system has emerged as a unique and advantageous technology. HPV is considered a major cause of cervical cancer, and timely detection and typing of HPV are crucial for risk prediction and management. A POCT detection system that can detect six high-risk HPV types within 40 minutes, with a sensitivity of 1 copy/μl (Liu et al., 2024). Additionally, an amplification-free CRISPR platform can detect HPV16 (Yu et al., 2023). HBV is closely related to the occurrence of liver cancer and can be transmitted through mother-to-child, blood, and sexual contact. A CRISPR-Cas12a-mediated SERS detection method was established, enabling the detection of HBV DNA at 0.67 pM within 50 minutes (Du et al., 2023). The application of the CRISPR-Cas12a system in DNA virus detection, especially in the detection of HPV and HBV, offering important insights for the development of future pathogen detection technologies. It is worth noting that when using Cas13 to detect DNA, the amplified products need to be transcribed into RNA by T7 (Tian et al., 2022). In the study, DNA amplified by RPA was reverse transcribed into RNA using T7 prior to reaction with CRISPR-Cas13 (Xu et al., 2024). Separately, a CRISPR-Cas14a-based cascade colorimetric detection method was established, capable of detecting ASFV as low as 5 copies/μL and distinguishing mutant ASFV DNA with a 2-nt difference (X. Zhao et al., 2024).

In summary, the application of CRISPR technology in the detection of viral pathogens has demonstrated its superiority in sensitivity, specificity, and rapid response, providing significant technical support for clinical testing and public health (Shokoohi et al., 2025b). With continuous technological advancements and optimizations, future CRISPR detection platforms are expected to detection more viruses.

### 3.2 Bacterial pathogens

The detection of bacterial pathogens holds significant importance. Among them, the rapid diagnosis of Mycobacterium tuberculosis is key to controlling the spread of tuberculosis. Recent studies have shown that combining CRISPR technology with isothermal amplification techniques can achieve rapid detection of Mycobacterium tuberculosis. For example, the multiplex cross-displacement amplification technique (CRISPR-MCDA) with the CRISPR-Cas12b system can complete the detection of Mycobacterium tuberculosis within 70 minutes, with a detection limit of 5 fg/μL (Yang et al., 2023).

In the detection of drug-resistant bacteria, CRISPR technology also shows excellent potential. For the detection of MRSA, using an amplification-free CRISPR and ECL to detect the mecA gene in MRSA, modifying the reporter molecules with Ag to make the electrochemical signal changes between positive and negative more pronounced (Suea-Ngam et al., 2021). A multifunctional CRISPR biosensor based on colorimetric, photothermal, and fluorescent signals (CPF-CRISPR) was developed for detecting MRSA, overcoming the limitation of single-signal output in existing biosensors (Zheng et al., 2023). An interesting OR-gated logic was adopted to detect MSSA, MRSA, E. coli, and hepatitis B virus through a cascade positive feedback amplification-free CRISPR detection system (Lim et al., 2025). This method includes T1 and T2 reactions. In the T1 reaction, crRNAs corresponding to the four pathogens are added. Whether it is a single pathogen infection or a mixed infection, the T1-CRISPR system can be activated and the target BNA in the T2 reaction system can be released through trans-cleavage, thereby triggering the release of T2-gDNA and forming a positive feedback signal amplification mechanism. This design significantly reduces detection costs and sample requirements, providing an efficient and economical solution for multiplex pathogen detection. These technologies can provide information on bacterial drug resistance, aiding in drug selection and infection control.

CRISPR technology has shown unique advantages in the detection of bacterial pathogens. It is therefore relevant for both improving the timely management of infections and for inspiring new approaches in clinical diagnostics.

### 3.3 Others pathogens

The application of CRISPR technology in detecting fungi and parasites has garnered increasing attention from researchers, especially in public health and clinical diagnostics. CRISPR enables rapid and accurate detection of specific fungi and parasites, supporting early diagnosis and treatment.

In the field of fungal detection, a method with a sensitivity of 30 CFU/mL was developed for Candida albicans, demonstrating 100-fold higher sensitivity than microscopic staining (Shen et al., 2024). This approach is rapid, device-free, and suitable for resource-limited settings. For malaria detection, a CRISPR-Cas12-based method was established that enables rapid identification of Plasmodium species within 20 minutes (Wei et al., 2023). For Trichomonas vaginalis, which causes vaginitis or urethritis, a CRISPR-based detection platform was created (Li et al., 2022d). Regarding Mycoplasma pneumoniae, a common respiratory pathogen, the CRAFT platform was developed, integrating nucleic acid extraction, RPA, CRISPR reaction, and signal readout to achieve rapid, sensitive, and specific detection (Li et al., 2025).CRISPR technology, both amplification-based and amplification-free, has been applied for pathogen diagnostics, as summarized in **Table 3**.

**Table 3:** Applications of amplification-based CRISPR and amplification-free CRISPR in the detection of different pathogens. .

Pathogen	Amplification method	Detection method	Sensitivity	Specificity	Time	References
HCV	LAMP	Fluorescence,LFA	10 ng/μL	100.00%	100 min	(Kham-Kjing et al., 2022)
O157:H7	LAMP	Fluorescence	9.2×10^0^ CFU/mL	100.00%	<60 min	(Wang et al., 2024)
Cps	LAMP	Fluorescence	10^2^aM	100.00%	<60 min	(Wang et al., 2023a)
C. jejuni	LAMP	Fluorescence	8 CFU/mL	100.00%	70 min	(Li et al., 2022a)
HPS	RPA	Fluorescence	0.163 pg/μL	96.60%	<60 min	(Zhang et al., 2023a)
AIV(H5)	RPA	Fluorescence,LFA	1.9 copies/μL l1.9×10^3^ copies/μL	100.00%	60-150min	(X. Zhou et al., 2024)
H. pylori	RPA	Fluorescence	1.4 copies/μL	100.00%	45min	(Li et al., 2023b)
TB	Amplification-free	gFET	2.42 × 10^−18^ M	98.60%	5min	(Wang et al., 2025)
Salmonella	Amplification-free	Cascade CRISPR	84 CFU mL	100.00%	30min	(Bao et al., 2023)
influenza A,HPV	Amplification-free	Digital CRISPR	10 copies/μL, 5 copies/μL	100.00%	75min	(Li et al., 2024)
SARS-Cov-2	Amplification-free	Cascade CRISPR	1 aM	100.00%	20 min	(Zhang et al., 2023c)
HPV16,HPV18	Amplification-free	ECL	0.4 fM, 0.51fM	100.00%	120 min	(Nguyen et al., 2025)

## 4 Discussion

Compared to traditional pathogens detection methods, CRISPR technology offers advantages in sensitivity and specificity. Amplification-based CRISPR methods achieve detection sensitivities as low as 1 copy/μL through amplification but may introduce false-positive results due to contamination risks associated with amplification steps (Li et al., 2023a). In contrast, amplification-free CRISPR strategies, while slightly less sensitive, generally exhibit superior specificity and avoid cross-contamination risks inherent in amplification-based approaches. In terms of operational workflow, amplification-based methods typically require 30–60 minutes and involve relatively complex procedures, whereas amplification-free strategies significantly reduce detection time due to simplified steps, with some assays completing in as little as 5 minutes (Wang et al., 2025). Currently, amplification-based CRISPR has been widely applied for detecting various pathogens, while amplification-free approaches remain in early stages for detecting fungi, parasites, and mycoplasmas, with their potential yet to be fully explored.

Although CRISPR technology demonstrates excellent performance in laboratory settings, its stability, reproducibility, and diagnostic accuracy in real-world clinical scenarios require further validation through large-scale, multi-center clinical studies, particularly for complex sample matrices such as blood, sputum, and stool. In terms of regulatory approval and commercialization, only a few platforms (e.g., SHERLOCK and DETECTR) have received Emergency Use Authorization (EUA) from the U.S. FDA (Gupta et al., 2021), while most CRISPR-based diagnostic solutions remain in the research and early translation stages. In terms of cost, the two strategies differ significantly in their cost structures. The primary expense of amplification-based CRISPR lies in the nucleic acid pre-amplification step (for instance, the reagent cost for isothermal amplification such as RPA or LAMP is approximately $3–6 per sample), and the reactions can be performed on standard PCR instruments, resulting in a low equipment barrier. Studies have shown that the cost per test for amplification-based CRISPR can be as low as $0.84 (Shen et al., 2020), yet the amplification step remains the major cost component. In contrast, while amplification-free CRISPR saves on reagent costs, it may incur higher expenses in design and equipment. For example, cascade CRISPR requires the design and synthesis of multiple crRNAs, increasing both design complexity and cost. Meanwhile, sensor-based CRISPR (e.g., SERS sensors) and digital droplet CRISPR rely on more expensive specialized equipment. Moreover nucleic acid extraction, device integration, and result standardization remain critical challenges for large-scale implementation (Sultana et al., 2024).

Future development could focus on simplifying sample preprocessing, improving device portability and automation, and enabling multiplex detection. The integration of microfluidic technology with CRISPR holds promise for combining nucleic acid extraction, amplification, and detection into a unified system, enhancing throughput and applicability, as demonstrated in the detection of Vibrio parasympathetics (Wu et al., 2021a). Furthermore, the emergence of portable detection devices (e.g., CRISPR platforms integrated with thermometer (Liu et al., 2022), and fully automated digital droplet systems (e.g., SATORI (Iida et al., 2023)) has further advanced the application of CRISPR technology in POCT. CRISPR technology is well-suited for pathogen diagnostics in different contexts, such as resource-limited or rapid on-site testing scenarios.
